# Replacement of Dietary Fish Protein With Bacterial Single Cell Protein Results in Decreased Adiposity Coupled With Liver Expression Changes in Female Danio Rerio

**DOI:** 10.21203/rs.3.rs-3044822/v1

**Published:** 2023-06-14

**Authors:** michael Williams, GEORGE B.H. GREEN, JOSEPH W. PALMER, CHRISTIAN X. FAY, SOPHIE B. CHEHADE, ADDISON L LAWRENCE, ROBERT J. BARRY, MICKIE L. POWELL, MELISSA L. HARRIS, STEPHEN A. WATTS

**Affiliations:** University of Alabama at Birmingham; University of Alabama at Birmingham; University of Alabama at Birmingham; University of Alabama at Birmingham; University of Alabama at Birmingham; Texas A&M University; University of Alabama at Birmingham; University of Alabama at Birmingham; University of Alabama at Birmingham; University of Alabama at Birmingham

## Abstract

**Background:**

Effective use of *Danio rerio* as a preclinical model requires standardization of macronutrient sources to achieve scientific reproducibility across studies and labs. Our objective was to evaluate single cell protein (SCP) for production of open-source standardized diets with defined heath characteristics for the zebrafish research community. We completed a 16-week feeding trial using juvenile *D. rerio* 31 days post-fertilization (dpf) (10 tanks per diet, 14 *D. rerio* per tank) with formulated diets containing either a typical fish protein ingredient or a novel bacterial SCP source. At the end of the feeding trial, growth metrics, body composition, reproductive success, and bulk transcriptomics of the liver (RNAseq on female *D. rerio* only with confirmatory rtPCR) were performed for each diet treatment.

**Results:**

*D. rerio* fed the SCP containing diet had body weight gains equivalent to the *D. rerio* fed fish protein, and females had significantly lower total carcass lipid, indicating reduced adiposity. Reproductive success was similar between treatments. Genes differentially expressed in female *D. rerio* provided the bacterial SCP compared to females given fish protein were overrepresented in the gene ontologies of metabolism, biosynthesis of cholesterol precursors and products, and protein unfolding responses.

**Conclusion:**

These data support the development of an open-source diet utilizing an ingredient that correlates with improved health profiles and reduced variability in notable outcomes.

## INTRODUCTION

Zebrafish (*Danio rerio*) have emerged as an increasingly important model organism for biomedicine and other scientific disciplines [1]. However, as the field matures, it is becoming increasingly apparent that the nutritional goals for optimal use of this model should evolve beyond basic production and reproduction to the establishment and maintenance of clinically healthy research subjects. We recognize that rigor and reproducibility in biomedical research have become a major focus at the NIH and within the research community at large [2]. Commercial diets used in the zebrafish research community lack ingredient transparency, standardization, and control of macro- and micro-nutrient composition. Feed variability has been shown to lead to variable outcomes among studies [3], yet researchers are often unable to mitigate these issues because quantitative and qualitative ingredient information is not readily available [4].

*D. rerio* growth is largely affected by dietary protein levels, with a positive linear correlation between weight gain and protein content when the latter is increased up to the predicted daily dietary requirement (approximately 38–45% of dry matter for juvenile *D. rerio* when fish meal is used as the protein source [5]). However, commercial diets typically contain 50 to 65% of dry matter protein obtained from a variety of sources (plant and/or animal). Excess protein can cause shifts in metabolic pathways that could be deleterious [6]. The quality and quantity of protein sources, other nutrients and anti-nutritional factors in commercial diets varies substantially among sources. Typical animal protein sources in fish diets include fish meal, squid meal, krill meal, casein, whey, gelatin, and feather and poultry meal [7]. Widely used plant sources include soy, wheat or corn products, cottonseed meal, and oats. Several plant-based protein sources currently used in *D. rerio* and Atlantic salmon diets (soy and gluten respectively) can contain or mimic anti-nutritional factors that lead to alterations in immune function [8, 9].

For most animal diets, use of multiple protein sources ensures that amino acid profiles and other associated bioactive compounds are sufficient to promote growth and health. Recent studies have introduced novel bacteria and yeast single cell protein (SCP) sources. Computer-supported SCP production technologies allows manufacturing of these protein sources in large quantities with high levels of purity and consistency [10]. SCP appears to be an excellent fish meal alternative in several food fish and other aquatic species, indicating a sufficient replacement of classical protein sources [11, 12]. SCP appears free of anti-nutritional factors, making SCP a higher value protein source than many derived from plants. To evaluate the value of SCP as a standardized fish diet, our study compares health outcomes (survival, weight gain, adiposity, and reproductive success) when varying dietary protein content, fat content, and replacement of traditional fish diet protein source (fish meal) for a bacterial SCP source. Furthermore, we employed transcriptomic analysis to interrogate whether substitution of SCP into the diet leads to changes in gene expression within the liver that could indicate positive or negative influences of SCP on metabolic health. Results from this study will assist in the creation of defined open-source diets for the zebrafish community.

## METHODS

### Experimental Housing and Husbandry

All procedures were approved by the UAB IACUC and adhere to standard *D. rerio* husbandry requirements for housing and euthanasia. *D. rerio* embryos (AB strain) were randomly collected from a mass spawning of males and females. Embryos were transferred to Petri dishes (n = 50 per dish) and incubated at 28.5°C until 5 days post fertilization (dpf). At 5-dpf, hatched larvae were polycultured in 6-liter static tanks (n = 240 larvae per tank) with the rotifer *Branchionus plicatilis* at a salinity of 4 ppt, and enriched with *Nannochloropsis* (RotiGrow Omega, Reed Mariculture, Campbell, CA, USA). At 11-dpf, all tanks were fed *Artemia* until 28 dpf. At 28 dpf, all 6-liter tanks were combined, and *D. rerio* randomly distributed into 2.8-L tanks at n = 14 fish per tank. Each tank was then randomly assigned to one of the dietary treatments (n = 10 tanks per treatment) and the 16-week feeding trial initiated. To obtain initial weights and lengths, a sub-sample of *D. rerio* (n = 50) were individually weighed and photographed prior to experiment implementation. For the first 2 weeks of the trial, *D. rerio* were provided a ratio of 10% of initial body weight per day of powdered feeds. Daily ratios were weighed for individual tanks. Rations were adjusted based on weight gain and food conversion ratios every two weeks. *D. rerio* were fed at 09.00 and 17.00 each day.

All tanks were maintained at approximately 28°C and 1500 μS/cm conductivity in a commercial recirculating system (Aquaneering, San Diego, CA, USA), with 5.4L exchanged from each tank per hour. Municipal tap water was passed through mechanical filtration (5μm sediment filter and charcoal), reverse osmosis, and a cation/anion exchange resin. Synthetic sea salts (Crystal Sea, Marine Enterprises International, Baltimore, MD, USA) were added to adjust conductivity for the system water source. Sodium bicarbonate was added as needed to maintain pH of the system water at 7.4. Total ammonia nitrogen, nitrite, and nitrate were measured colorimetrically once weekly. To help sustain adequate water quality, a minimal water exchange of 20% was performed on the recirculating system once per week. Tanks were maintained on the same recirculating system throughout the duration of the experiment. To reduce environmental confounding effects from noise, light, vibration, or other unidentified sources, tanks were cleaned and returned to a new position on the recirculating rack system every two weeks. Tanks were siphoned every other day to remove any excess uneaten feed or debris. Experimental animals were maintained under a 14-hour light/10-hour dark cycle with lights turned on at 07:00 local time. At the end of the study, *D. rerio* were euthanized by rapid submersion in ice-cold water for a minimum of ten minutes after opercular motion had ceased. Carcasses were stored at −80°C until analysis.

### Diet Preparation

Each diet contained cholesterol, menhaden oil, corn oil, vitamin (custom vitamin mixture MP Biomedicals, Santa Ana, CA, USA) and mineral premixes (MP Biomedicals 290284), and alginate binders (ingredients and catalog numbers listed in Table 1). Diets were identical in all nutrient profiles and ingredients adjusted to provide comparable lipid (n6:n3 ratios) and total amino acid content. Protein sources were casein (MP Biomedicals, Cat. no 0296012805) and either fish protein hydrolysate (Cat. no CPSP90, Scoular, Omaha, NE, USA) or single cell protein sources provided by Meridian Biotech (The Woodlands, TX, USA) consisting of bacteria (composition in Table 2, 3, and 4). All ingredients were weighed on an analytical balance (Mettler Toledo New Classic MF Model MS8001S or Model PG503-S Mettler-Toledo, LLC. Columbus, OH, USA) and mixed using a Kitchen Aid Professional 600 Orbital Mixer (Kitchen Aid, Benton Harbor, MI, USA). The diets were cold extruded into strands to preserve nutrient content using a commercial food processor (Cuisinart, East Windsor, NJ, USA) and strands were air-dried for 24 hours on wire trays. Diets were labelled as SR (standard reference diet that contains fish protein hydrolysate) and BP (bacterial single cell protein containing diet).

### Growth and Body Composition Parameters

Following random assignment of tanks to the dietary treatments, *D. rerio* were weighed together as a group from each treatment tank to 0.001g and photographed from above in a clean 1-L breeding tank using a D70 camera (Nikon, Tokyo, Japan) every two weeks throughout the experiment. At termination of the feeding trial, all *D. rerio* were sexed and weighed individually to 0.001g and photographed. All photographs were analyzed with NIS Elements 3.1 software to determine standard length (measured from tip of snout to the distal end of the caudal peduncle) to 1mm. Total lipid for females (n = 10) and males (n = 9–10) of each diet was determined using the Folch lipid extraction protocol optimized for *D. rerio*.

### Egg Production and Viability

At the end of the 16-week feeding trial males and females from each diet treatment were separated, placed in different 2.8-L tanks at n = 10 and maintained on the on the treatment diets an additional 4 weeks for breeding analysis. Maintenance conditions and feeding regime continued as described. For each diet, egg production and embryo viability (at 4 and 24 hours post fertilization (hpf)) were assessed. Females and males were randomly selected from each tank and paired with *Artemia*-fed females and males from the UAB Aquatic Animal Resource Core. Breeding pairs (1 male and 1 female) were transferred to 1-L breeding tanks (Aquaneering) with a divider separating the pair on the evening prior to breeding. Dividers were removed when the lights were turned on the following morning for a two-hour period of spawning, after which each male and female were returned to their respective tanks. Successful spawning was recorded and females from unsuccessful spawning events were removed from the study and euthanized as described below. Immediately after spawning, eggs/embryos from successful breeding pairs were collected, cleaned, counted, and scored as viable embryos or non-viable eggs. After counting, viable embryos were divided into Petri dishes (n = 50) and incubated overnight at 28.5°C in fresh Embryo Medium (1500 μS/cm conductivity). At 24 hpf, viable embryos were counted and assessed for normal development based on their morphology. The 10 random breeding pairs for each diet were set up once every other week for four weeks with females bred twice and males once, resulting in 17–20 total breeding events per diet for females and 9–10 breeding events for males.

### RNA isolation

At termination of the feeding trial, livers from 5–7 males and 8 females from each dietary treatments were dissected out, flash frozen in nitrogen and transferred to −80°C for storage. Subsequently, RNA was isolated from these livers using RNeasy Lipid Tissue Mini Kit (Qiagen) per the manufacturer’s instructions. Purified RNA was subjected to quantification and purity assessment via NanoDrop.

### RNA sequencing and analysis

Four female liver RNA samples from each dietary treatment were sent to the UAB Genomics Core Laboratory, Heflin Center for Genomic Sciences (Birmingham, AL, USA). From this RNA, poly-A selected indexed RNA libraries were prepared using the Ultra II RNA Library Prep kit and sequenced on the Illumina NextSeq 500 platform to achieve a minimum of 30 million, single-end, 75bp reads per sample. Bioinformatic analysis of the RNA sequencing reads were evaluated for sequence quality using FastQC (ver. 0.11.8). On average, the Phred quality score across samples was greater than 35 indicating a high level of confidence in the accuracy of individual base calls (> 99.9%). Next, the sequences were aligned to the Ensembl *D. rerio* reference genome (GRCz11) and individual gene counts were obtained for each sample using the quantMode feature of the STAR aligner (ver. 2.5.2b). Differential expression analysis was performed using DESeq2 (ver. 1.22.2) and methods similar to those published previously [13]. Gene Ontology (GO) analysis (http://geneontology.org, accessed 24th March 2023) analysis was performed on differently expressed genes (DEGs) determined via DESeq2 (ver. 1.22.2) [14–16]. The DeSeq analysis was run utilizing the default setting and produced a full list of genes that can be found in Supplemental Data 1. The data was filtered to obtain a concise list of highly differentially expressed genes, using the follow criteria: BaseMean > 500, log2FC > |1.5|, and a *p*-value < 0.1. A number of DEGs with a BaseMean < 500 and relating to oocyte biology and oocyte metabolism were observed and these are attributed to potential oocyte contamination during liver dissections. The liver and oocytes in female fish are proximal to each other anatomically. Filtering based off the parameters discussed above.

### Real-Time PCR

For Real-Time PCR analysis, a High-Capacity cDNA Reverse Transcription Kit (Applied Biosystems, Foster City, CA) was used to synthesized cDNA as per manufacturer’s instructions with a starting amount of 5ug of total RNA in a 100uL reactions run on a SimpliAmp^™^ Thermal Cycler (Applied Biosystems). 5uL of cDNA was diluted to 1:20 and used for a 20uL total reaction using TaqMan^™^ Fast Advanced Master Mix (Applied Biosystems) and MicroAmp^™^ Fast Optical 96-Well Reaction Plates (Applied Biosystems). Gene specific TaqMan primers were purchased from Applied Biosystems and were designed by the manufacturer (*b2m*-Dr03432699_m1, *rpl7*-Dr03114687_g1, *ldlra*-Dr03109730_m1, *pdia4*-Dr03080709_m1, *hspa5*-Dr03107861_m1, and *hmgcs1*-Dr03107117_m1). 40 rt-PCR cycles were run on a QuantStudio 3 Real-Time PCR System and results analyzed with QuantStudio^™^ Design & Analysis Software v1.5.1using (Applied Biosystems). *b2m* and *rpl7* as were used for normalization by geometric average [17].

### Statistical Modeling and Analysis

Data from this study were analyzed with RStudio Statistical Software (R Core Team, 2016, v0.99.896), and graphs generated with Statistical Package for Social Science (SPSS) ver.2.3 (IBM, Armonk, NY). All data were analyzed for normality and equal variances. Any datasets with a non-normal distribution were log-transformed. All terminal analyses for continuous outcomes were stratified by sex. Terminal wet body weight, and total body length were compared separately by linear random effects model with tank as a random variable. Total body moisture was analyzed by ANOVA, and fat mass was analyzed with ANCOVA, adjusting for dry body weight as a covariate. Since unequal variances were observed in rtPCR results, these data were analyzed using one-tailed Welch’s unequal variances t-test to validate RNAseq. For total embryos produced, previous examination of similar datasets revealed over-dispersion with excessive truncated zeroes (non-successful breeding events), indicating that these data were well-suited for a hurdle negative binomial model [18]. Data for total embryo production were fitted to a hurdle negative binomial model with help of the pscl package of the R language [19]. Diet and week of breeding were included as predictors in the model and analyzed for main effects on total embryo production. The outcome for embryo viability is a proportion between 0 and 1, with two types of zeroes present: truncated (non-successful breeding events) and sampling (zero viable embryos produced). For this reason, a zero-inflated beta regression model (BEZI) is selected as the most appropriate model. The first component of the BEZI model uses logistic regression and the parameter nu (controls the probability that a 0 occurs) to analyze the zero counts and determine the probability of 0 viable embryos produced. The second component analyzes the positive counts by fitting a beta regression to compare the expected proportion of viable embryos and includes the parameters mu (mean) and sigma (variance) (John Dawson, Dept. of Biostatistics, personal communication). The best fit model usually includes all three parameters, and is selected with help of the gamlss package of the R language [20].

## RESULTS

**Figure 1:**
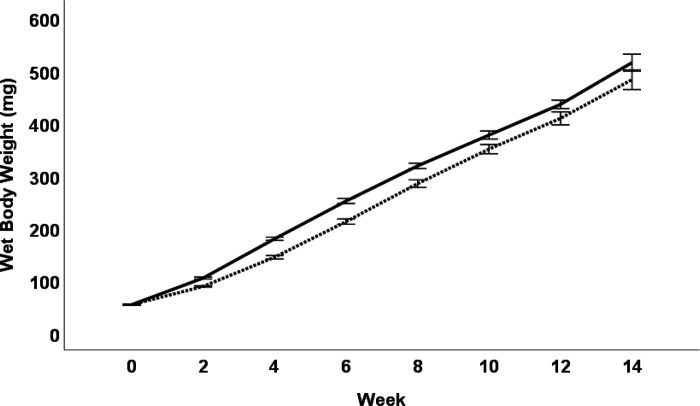


**Figure 2:**
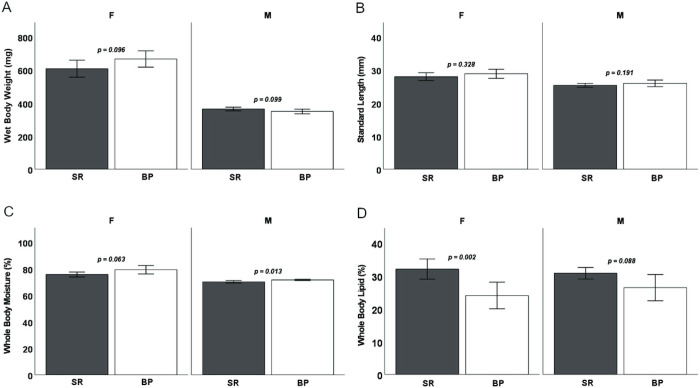


**Figure 3:**
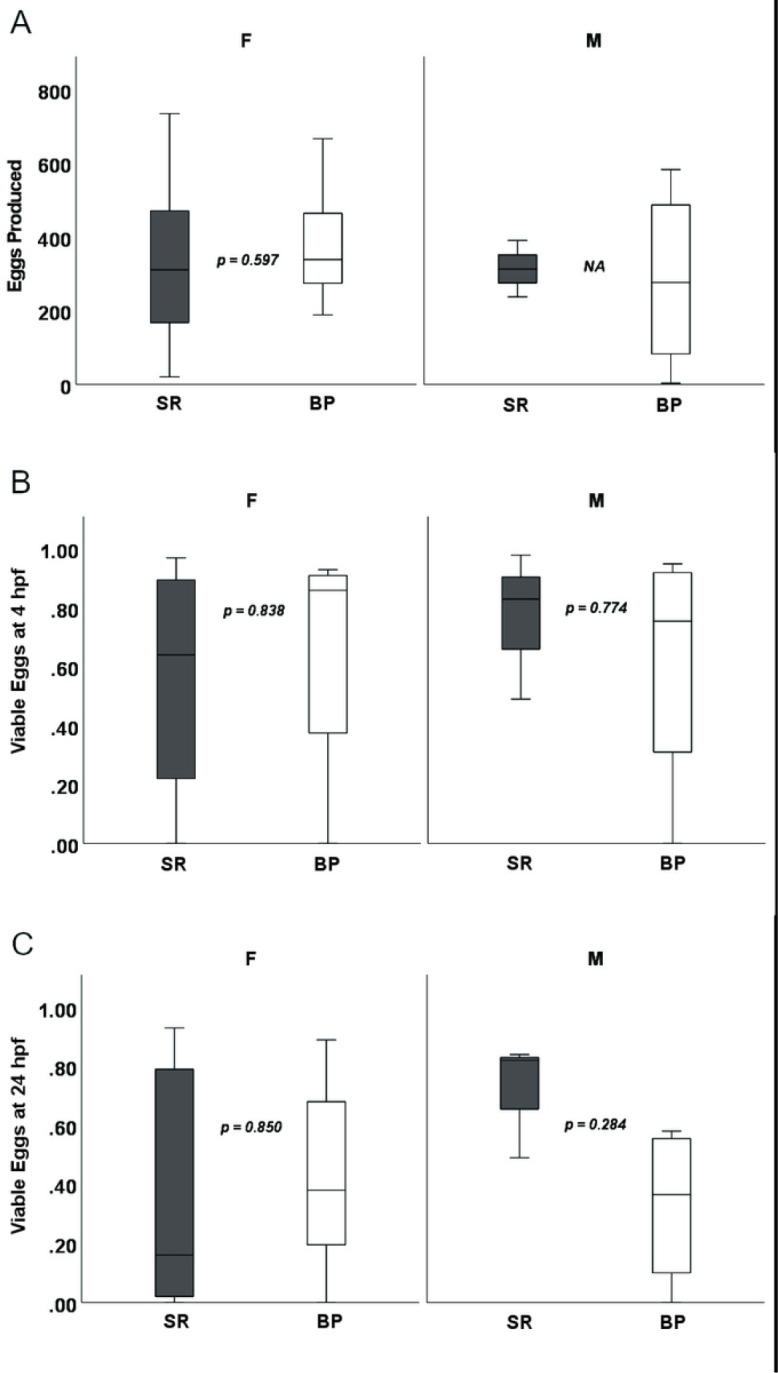


Both diets sustained *D. rerio* growth and development over the 16-week feeding trial (Fig. 1.). For terminal wet body weights, when sexes were separated, males and females showed no differences in terminal body weight (*p* = 0.099 and *p* = 0.096 respectively) (Fig. 2A). Male and female *D. rerio* fed the SR and BP diets showed no significant differences in standard body length (*p* = 0.191 and *p* = 0.328 respectively) (Fig. 2B). Females had no significant differences in total body moisture (*p* = 0.063), but males fed the BP had a higher total body moisture compared to males fed the SR diet (*p* = 0.0129) (Fig. 2C). For total body lipid, female *D. rerio* fed the BP diet had less adiposity than *D. rerio* fed SR (*p* = 0.002), but males show no significant difference (p = 0.088) (Fig. 2D). In regard to reproduction, there were no differences in spawning success for males and females fed the diet treatments paired with the opposite sex fed *Artemia* (*p* = 0.900, *p* = 0.597, respectively; Table 5). Total egg production between female *D. rerio* fed the SR and BP diets were not significantly different (*p* = 0.597, Fig. 3A). Eggs fertilized by males and females fed the BP diet had no significant differences in viability than those fed the SR diet at either 4 hpf (*p* = 0.774 and *p* = 0.838 respectfully) (Fig. 3B) or 24 hpf (*p* = 0.284 and *p* = 0.850 respectfully) (Fig. 3C). For female egg viability the first or second breeding event had no impact on egg viability at either 4 or 24 hpf (*p* = 0.598 and *p* = 0.199 respectively).

**Figure 4:**
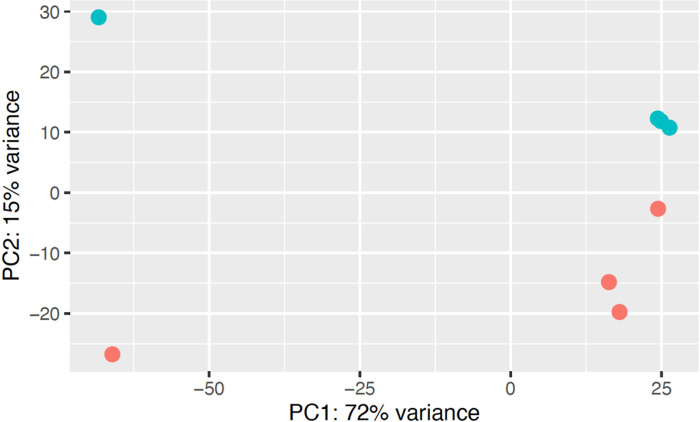


**Figure 5:**
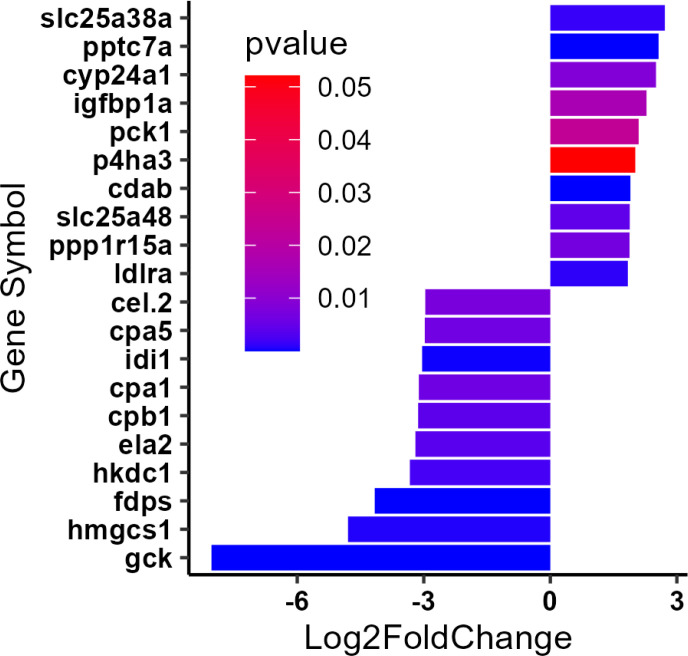


**Figure 6:**
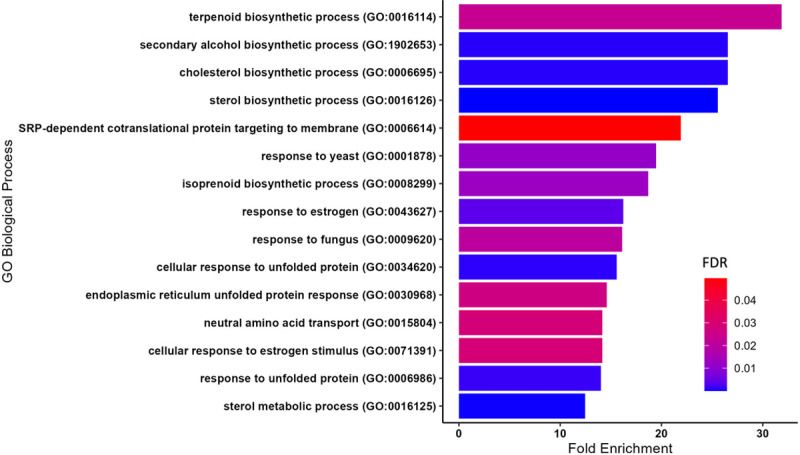


**Figure 7:**
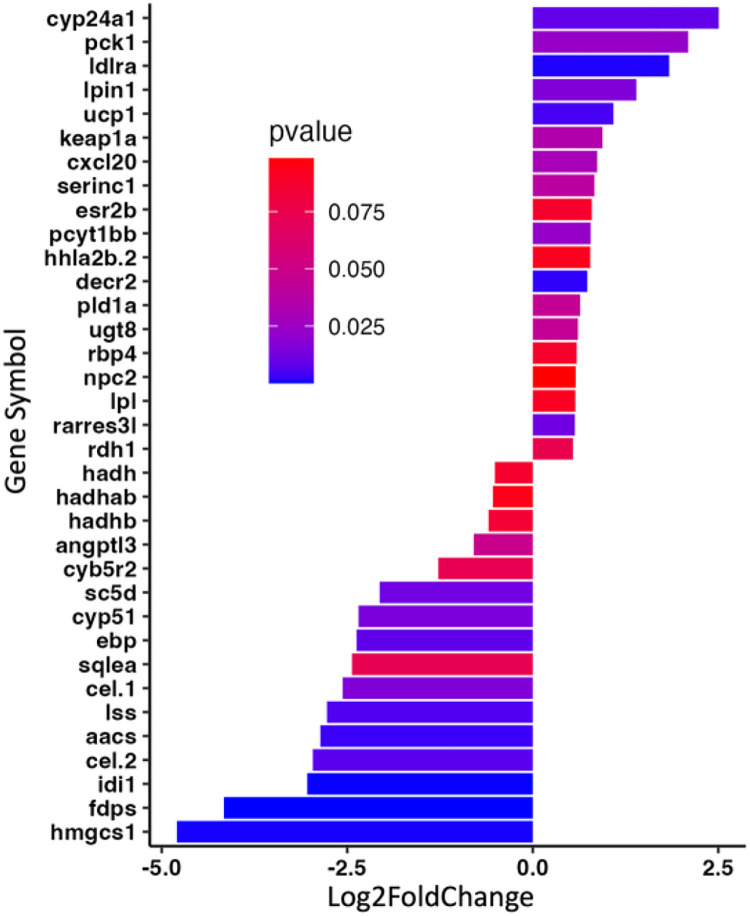


In order to evaluate molecular changes specifically associated with a complete substitution of fish protein hydrolysate (SR diet) with bacterial single cell protein (BP diet), we assessed changes in liver transcriptomics of female *D. rerio* using RNAseq. Principal component analysis of the regularized log transformed count values (rlog) for each sample revealed that the majority of the biological replicates within an individual diet clustered together, and the differences between the diets was explained by the variance observed across principal component 2 (PC2, 15%). Two samples from each condition exhibited unusual variance across PC1 (72%), however, since these two samples clustered with their diet as expected across PC2, they were not excluded from the analysis (Fig. 4). Comparing the normalized gene expression between *D. rerio* fed either the SR or the BP diet revealed 267 differently expressed genes (DEGs; BaseMean > 500, log2FC > |1.5|, and a p-value < 0.1). The top 10 up- and downregulated genes are presented in Fig. 5. Using GO analysis on all 267 DEGs, we determined key biological pathways associated with these genes. The top 15 pathways were selected based off the largest fold enrichment, and a False Discovery Rate (FDR) < 0.05 (Fig. 6). A complete table of all GO pathways outputted are presented in Supplementary Data 2. We found that the majority of these 267 DEGs clustered into major biological processes such as: terpenoid biosynthetic process (GO:0016114; FDR = 2.35E-02) (gene ratio 3/11), secondary alcohol biosynthetic process (GO:1902653; FDR = 7.21E-04) (gene ratio 5/22), sterol biosynthetic process (GO: 0016126; FDR = 1.74E-05) (gene ratio 7/32), steroid biosynthetic process (GO: 0006694; FDR = 6.86E-04) (gene ratio 7/66), and cellular response to unfolded protein (GO: 0034620; FDR = 9.13E-04) (gene ratio 6/45). Our GO analysis included several lipid related pathways, which may reflect the decrease in adiposity observed amongst female *D. rerio* fed the BP diet. These included: sterol biosynthetic bioprocess (GO: 0016126; FDR = 1.74E-05) (gene ratio 7/32), “cellular response to lipid (GO:0071396; FDR = 3.65E-03) (gene ratio 7/159 fatty acid metabolic process (GO: 0006631; FDR = 1.30E-02) (gene ratio 5/57), “sterol metabolic process (GO:0016125; FDR = 1.69E-04) (gene ratio 7/32), lipid metabolic process (GO:0006629; FDR = 2.50E-06)” (gene ratio 28/873), “fatty acid catabolic process (GO: 0009062; FDR = 2.16E-02)” (gene ratio 5/57), and “lipid biosynthetic process (GO:0008610; FDR = 9.25E-04)” (gene ratio 15/415). Genes involved in these pathways can be seen in Supplementary data 3 (Fig. 7).

**Figure 8:**
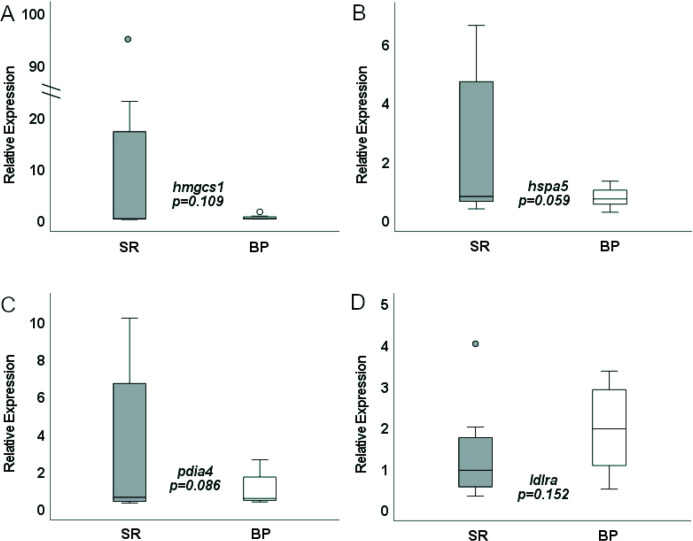


Validation of the RNAseq data was performed using quantitative PCR and tested DEGs related to two of the major GO pathways: cholesterol homeostasis genes *hmgcs1* and *ldlra*, and the unfolded protein response genes *hspa5* and *pdia4* (Figs. 7 and 8). Expression level of *hmgcs1, pdia4*, and *hspa5* in female *D. rerio* given either the SR or BP diet all trended in the same direction as seen in the transcriptomic analysis with decreased expression of all three genes in the BP fed females compared to the SR fed females. However, sample variation was high and these differences in expression were only significant at an alpha near 0.1 (*p* = 0.109, 0.059, and 0.086 respectively). Expression of *ldlra* in female livers again was in the same direction as detected by RNAseq with increased expression in BP fed females compared to SR fed females, yet high sample variation led to differences that were not significant (*p* = 0.152). Notably, unlike the transcriptomic and quantitative PCR results observed in female *D. rerio*, expression of *hmgcs1*, *pdia4*, and *ldlra* were not differentially expressed in male *D. rerio* fed the same diets suggesting an impact of diet by sex effects (*p* = 0.217, 0.172, and 0.303 respectively). Male *hspa5* expression difference was not in the same direction as what was seen in the transcriptome analysis and was therefore excluded for the one-tailed analysis.

## DISCUSSION

For single cell proteins, it is notable that total replacement of a high-quality fish protein hydrolysate with bacterial SCP fully supported growth metrics of body mass weight gain and total length. Markers of fecundity were also fully supported by bacterial SCP. Remarkably, the relative lack of adiposity coupled with concomitant weight gain suggests that females fed bacterial SCP maintained or increased body lean matter (fat free mass), the majority of which is most likely protein [21]. Similar trends were observed in males. In addition to the body composition changes, in the livers of these *D. rerio* we observed DEGs associated with gene ontologies related to metabolism (GO190263, GO0015804), biosynthesis of cholesterol precursors and products (GO0016125, GO0006695, GO0016126, GO0008299), and unfolded protein responses (GO0034620, GO0030968, GO000692653) [14, 15].

The body composition changes observed are related to gene expression changes in gene ontologies associated with metabolism. A substantial number of DEGs related to lipid metabolism were present in our most altered gene ontologies via diet in female livers. Hamidoghli et al. [22] evaluated the impact of bacterial SCP in replacing fish meal in whiteleg shrimp *Litopenaeus vannamei*, and observed similar body composition outcomes, including an increased crude protein and decreased crude lipid in tail muscle after a 9-week feeding trial. Replacement of fish meal with bacterial SCP in the rainbow trout *Oncorhynchus mykiss* resulted in increased weight gain over a 60 day feeding trial at a 50% replacement, but limited weight gain at 100% replacement (which authors attributed to a reduced feed intake related to palatability) [23]. Muscle analysis of the 100% SCP replacement fed O. *mykiss* correlated with decreased crude protein, lipid content, and n3/n6 fatty acid ratio. Additionally, SCP inclusion decreased enzyme activity related to digestion, including bile salt-activated lipase, which is important for TAG (triglycerides) and cholesterol absorption. These metabolic changes these authors observed could potentially have contributed to observed differences in body composition. The Nile tilapia *Oreochromis niloticus* fed diets with yeast SCP replacement of fish meal had decreased carcass lipid content with no differences in weight gain or carcass protein in a 12 week feeding trial [24]. Consequently, these data suggest that changes in gene expression related to lipid metabolism are associated with decreased whole-body lipid content without weight (fat free mass) loss, and is a positive attribute of fish meal replacement with SCP products.

Several of the gene ontologies altered by the SCP diet are related to cholesterol metabolism. Single cell proteins of bacterial origin have been reported to have prebiotic and probiotic properties, both of which influence cholesterol homeostasis and related genes via bile acid biosynthesis pathways [25, 26]. *Hmgcs1*, which was down regulated in livers of female *D. rerio* fed the bacterial SCP, is a part of the cholesterol biosynthesis pathway in *D. rerio* and other animals [27]. Oczkowicz et al. [28] found that pigs fed a single cell protein derived from corn dried distiller’s grains (cDDG) exhibited a decrease in expression of *hmgcs1* (mammalian *hmgc1* is homologous in *D. rerio* [29]). *ldlr* was upregulated in female *D. rerio* fed the SCP diet, and is responsible for the uptake of LDL cholesterol particles from blood circulation into organ tissues [30]. We hypothesize upregulation of *ldlr* will lower cholesterol in blood circulation in *D. rerio* and this may be compensatory to lower *de novo* cholesterol production.

SCPs contain beta-glucan, a potent prebiotic. Beta-glucan decreased circulating total cholesterol, HDL, LDL, and triglycerides and increased the HDL:total cholesterol ratio in rats [31]. Rats provided beta-glucan or spent brewers yeast diets also had lower liver total cholesterol with no differences in weight from rats fed a standard commercial feed. Carneiro et al. [32] replaced dietary fish meal (5% of the total diet) for *D. rerio* with a SCP composed of microalgae. This resulted in higher body weight gain over a 60-day feeding period with lower triglycerides, LDL, and total cholesterol and increased HDL. Combined, these studies suggest that *D. rerio* is a novel model for the study of cholesterol metabolism and its impact on liver health, and could be an excellent model for developing pre-clinical treatment and preventative strategies such as those provided by the use of statins [33]. Continued use of the *D. rerio* model can have a profound impact on our understanding of macronutrients in regulating health benefits.

In addition to changes in lipid and cholesterol metabolism, the expression of endoplasmic reticulum unfolded protein response genes was also influenced by inclusion of SCP in the diet. Unfolded protein responses are highly conserved among mammals and teleost species [34]. *Pdia4* and *hspa5* exhibited decreased liver expression in female *D. rerio* fed the bacterial SCP. *Pdia4* modulates inflammatory responses related to insulin signaling in a mouse model of genetic insulin resistance fed a high fat diet [35]. *Hspa5* expression in *D. rerio* was increased on a high fat diet and high fat diets with supplemented cholesterol [33]. The expression changes were concomitant with increased TAG and free cholesterol in the liver. *Pdia4* and *hspa5* have also been shown to have increased liver expression in mice with normal insulin sensitivity on a high fat diet [36, 37]. The altered expression of genes in this unfolding protein response ontology suggests that the bacterial SCP diet impacts ER stress commonly seen with obese phenotypes [38]. Future work is needed to determine lipid and cholesterol metabolism changes and their relation to animal health and SCP diets.

As suggested previously, bacterial SCP is an effective substitute for fish protein hydrolysate, and may positively influence physiological outcomes due to its comparable amino acid content. Bacterial SCP may also function as a prebiotic and/or probiotic. We speculate that physiological and transcriptional effects measured in the current study have a corroborative basis in the gut microbiome. It is possible that dietary effects are mediated by the gut microbiome, exerting microbial influences through altered nutrient processing, allocation, and signaling. Future work will focus on the overall understanding of the interactions of the dietary macronutrients, the microbiome and resulting metabolome, resource partitioning, and the gut-brain signaling axis. The zebrafish model shows great promise in elucidating changes in fundamental metabolic networks underlying dietary influence on tissue and organismal health. These data put us one step closer to the goal of establishing alternative novel protein ingredients for open-source diets for use in the *D. rerio* model, increasing the utility of this species as a pre-clinical research model.

## Figures and Tables

**Figure 9:**
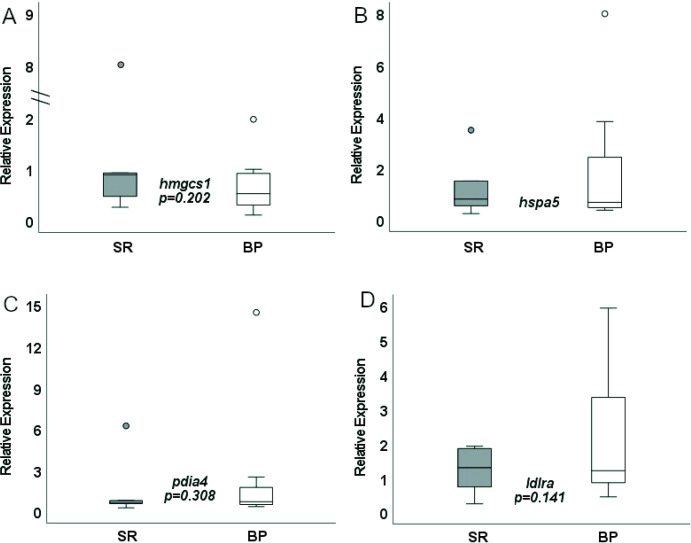


**Table 1:** Ingredient vendors and catalog numbers.

Ingredient	Vendor	Catalog Number
fish protein hydrolysate	The Scoular Company	CPSP90
dextrin type III	MP Biomedicals	0216005790
mineral mix AIN93G	MP Biomedicals	0296040002
casein low trace metals	MP Biomedicals	0296012805
soy protein isolated	MP Biomedicals	0290545605
corn oil	MP Biomedicals	0290141401
safflower oil	MP Biomedicals	0210288890
menhaden fish oil	Omega Protein	Virginia Prime Gold
vitamin diet fortification mixture	MP Biomedicals	0290465401
diatomaceous earth, acid washed	Andwin Scientific	D3877
alphacel non nutritive bulk	MP Biomedicals	0290045305
D-(+)-glucosamine hydrochloride	MP Biomedicals	0210178225
cholesterol NF	MP Biomedicals	02101380-CF
lecithin, soy, refined	MP Biomedicals	0210214790
ascorbyl palmitate	MP Biomedicals	0210078180
potassium phosphate monobasic	MP Biomedicals	02195453.5
wheat starch	MP Biomedicals	0290295225
alginate	TIC Gums	TICA-Algin 400
betaine	MP Biomedicals	150461
canthaxanthin	DSM	Carophyll Red

**Table 2:** Composition of diets used for feeding trial.

Ingredients g/kg	SR	BP
casein - low trace metals	350.00	350.00
fish protein hydrolysate	200.00	0.00
MRD Pro Batch 2	0.00	317.90
wheat starch	56.50	56.50
dextrin type III	16.10	16.10
alpha cellulose	10.00	10.00
diatomaceous earth	125.70	0.00
menhaden fish oil (ARBP) Virginia Prime Gold	26.00	39.00
safflower oil	45.50	40.30
alginate	20.00	20.00
soy lecithin (refined)	40.00	40.00
Vit Pmx (MP Vit Diet Fortification Mixture) ^A^	40.00	40.00
mineral Pmx aka premix (AIN 93G) ^B^	30.00	30.00
canthaxanthin (10%)	23.10	23.10
potassium phosphate monobasic	11.50	11.50
glucosamine	2.50	2.50
betaine	1.50	1.50
cholesterol	1.20	1.20
ascorbylpalmitate	0.40	0.40
Total	1000.00	1000.00

A. MP Biomedicals 904654: Vitamin A Acetate (500,000 IU/gm) 1.80000, Vitamin D2 (850,000 IU/gm) 0.12500, DL-a-Tocopherol Acetate 22.00000, Ascorbic Acid 45.00000, Inositol 5.00000, Choline Chloride 75.00000, Menadione 2.25000, p-Aminobenzoic Acid 5.00000, Niacin 4.25000, Riboflavin 1.00000, Pyridoxine Hydrochloride 1.00000, Thiamine Hydrochloride 1.00000, Calcium Pantothenate 3.00000, Biotin 0.02000, Folic Acid 0.09000, Vitamin B12 0.00135, measures are mg/g

B. AIN 93 mineral mix for Envigo (Indianapolis, IN): Sucrose, fine ground 209.496, Calcium Carbonate 357.0, Sodium Chloride 74.0, Potassium Phosphate, monobasic 250.0, Potassium Citrate, monohydrate 28.0, Potassium Sulfate 46.6, Magnesium Oxide 24.3, Manganese Carbonate 0.63, Ferric Citrate 6.06, Zinc Carbonate 1.65, Cupric Carbonate 0.31, Potassium Iodate 0.01, Sodium Selenite 0.0103, Chromium Potassium Sulfate, dodecahydrate 0.275, Lithium Chloride 0.0174, Boric Acid 0.0815, Sodium Fluoride 0.0635, Nickel Carbonate Hydroxide, tetrahydrate 0.0318, Ammonium Meta-Vanadate 0.0066 measures are mg/g

**Table 3:** Amino acid content of protein sources (as fed)

	SR	BP
Aspartic acid	6.09	4.69
Threonine	2.16	1.55
Serine	3.29	1.58
Glutamic acid	9.72	6.96
Proline	4.37	1.98
Glycine	7.68	2.85
Alanine	5.10	4.81
Cystine	1.17	0.29
Valine	3.88	3.18
Methionine	2.12	0.96
Isoleucine	2.92	2.31
Leucine	5.66	4.02
Tyrosine	5.26	1.60
Phenylalanine	3.06	2.14
Lysine	6.20	2.86
Histidine	1.71	0.64
Arginine	5.99	2.04
Tryptophan	0.70	0.70

**Table 4:** Macronutrient and energy content

	SR	BP
Calculated Protein Level (%) As Fed	47.29	50.03
Calculated Protein Level (%) Dry	52.54	55.59
Calculated Lipid Level (%) As Fed	11.04	10.98
Calculated Lipid Level (%) Dry	12.27	12.20
Calculated Soluable Digestable Carbohydrate Level (%) As Fed ^A^	25.28	31.24
Calculated Energy Level (cal/g) As Fed	4326	4714
Protein : Energy Ratio as fed	0.645	0.660
Ash (%) As Fed	15.06	5.91
Fiber (%) As Fed	1.33	1.83

A. Calculation used for soluble digestible carbohydrate: carbohydrate =100 − (protein % + fat % + ash %, + fiber %).

**Table 5:** Success of male and female breeding events. Attempts are pairings of males from the diet study with stock females or females from the diet study with stock males. Success Breeding represents bred pairs that resulted in eggs being released.

Male	Success Breeding	Attempted Breeding
SR	3	9
BP	3	10
Female		
SR	12	20
BP	7	20

**Table 6:** Major genes upregulated and downregulated based off the top 5 up and downregulated genes (baseMean > 500, loq2FoldChanqe > 1.5, p-value < 0.5).

Gene Name	symbol	baseMean	log2FoldChange	pvalue	Gene Name
ENSDARG00000103277	cyp24a1	2088.71751	2.505391699	0.00862172	cytochrome P450, family 24, subfamily A, polypeptide 1
ENSDARG00000013522	pck1	10429.6517	2.094444246	0.02329048	phosphoenolpyruvate carboxykinase 1 (soluble)
ENSDARG00000029476	ldlra	2368.11396	1.83732496	0.00097603	low density lipoprotein receptor a
ENSDARG00000020239	lpin1	1218.46971	1.395904305	0.01583396	lipin 1
ENSDARG00000023151	ucp1	41267.5844	1.086290394	0.00453535	uncoupling protein 1
ENSDARG00000103025	hmgcs1	9077.07414	−4.79608537	0.00056027	3-hydroxy-3-methylglutaryl-CoA synthase 1 (soluble)
ENSDARG00000040890	fdps	2581.87754	−4.162776475	1.0552E-05	farnesyl diphosphate synthase (farnesyl pyrophosphate synthetase, dimethylallyltranstransferase, geranyltranstransferase)
ENSDARG00000019976	idi1	1169.73266	−3.03909491	0.00014978	isopentenyl-diphosphate delta isomerase 1
ENSDARG00000029822	cel.2	2031.2092	−2.963990299	0.00737772	carboxyl ester lipase, tandem duplicate 2
ENSDARG00000012468	aacs	701.360642	−2.861630648	0.00287917	acetoacetyl-CoA synthetase

## Data Availability

Bulk RNA sequencing datasets of *D. rerio* samples are publicly available on the BioSample Submission Portal (https://www.ncbi.nlm.nih.gov/bioproject/) under the BioProject ID PRJNA973118. Additional data sets are available from authors upon request.
